# A Reconfiguration Strategy of Distribution Networks Considering Node Importance

**DOI:** 10.1371/journal.pone.0168350

**Published:** 2016-12-19

**Authors:** Juan Wen, Yanghong Tan, Lin Jiang

**Affiliations:** 1 College of Electrical and Information Engineering, Hunan University, Changsha, Hunan, China; 2 College of Electrical and Information Engineering, University of south China, Hengyang, Hunan, China; Tianjin University, CHINA

## Abstract

Node importance degree is a vital index in distribution network reconfiguration because it reflects the robustness of the network structure by evaluating node importance. Since the traditional reconfiguration ignores this index, the reconstructed network structure may be vulnerable which would reduce the security and stability of the distribution systems. This paper presents a novel reconfiguration strategy considering the node importance. The optimization objectives are the improvement of the node importance degree and the reduction of power loss. To balance the objectives, the reconfiguration mathematical model is formulated as a compound objective function with weight coefficients. Then the quantum particle swarm algorithm is employed to address this compound objective optimization problem. The strategy can model different scenarios network reconfiguration by adjusting the weight vector based on the tendencies of the utility decision maker. Illustrative examples verify the effectiveness of the proposed strategy.

## Introduction

As one of the main findings in the study of complex systems, the ubiquitous presence of networks in nature and society has attracted much attention of many researchers. The topologies of such networks have been profusely researched and some fundamental structures have been discovered [1]. These network topologies are widely employed to analyze the characteristics of complex real systems, such as electrical power systems [2–5], biomedicine systems [6–11], communication systems [12], traffic systems [13], and so on. This research interests in the power distribution networks.

In a distribution system, many feeders are interconnected with the switches. These switches are divided into two types of switches: sectionalizing switches and tie switches. By changing the status of the switches, power loads can be transferred from one feeder to another feeder. That is, the network topology can be reconfigured to improve distribution system reliability, economy and security. Under normal or abnormal operating conditions, network reconfiguration is a process that consists in altering the status of sectionalizing and tie switches. We obtain an optimal radial topology which satisfies desired objectives of balancing overloads [14], minimizing power loss [15], and improving voltage profile [16]. Traditional goal of the power loss minimization has gained much attention because excessive power loss would lead to high operating cost [15]. Other objectives are to increase the system reliability and usage life of electrical equipments [17–19]. With the growing complexity of the distribution systems, the node importance of the network is becoming more and more important because it reflects the robustness of the network structure.

An effective evaluation index of node importance is node importance degree which is applied to evaluate the robustness and survivability of power networks [20–27]. Researchers have explored the fundamental definition of node importance degree. Watts and Strogatz firstly reveal that the power system of the western United States is a small-world topology network. In this topology network, they suggest the node importance degree based on the complex theory is an index to evaluate the network robustness [20–21]. References [22–23] give the computational methods of node importance degree from the perspective of system science. And then it is used to analyze the relation of the nodes and cascade failures in [24]. Considering the topological characteristics of scale-free networks, the node importance degree is regarded as a reconfiguration index which is used to evaluate the performance of transmission line network reconfiguration [25–26]. Reference [27] adopts the maximization restoration paths as the reconfiguration objective to find an optimal restoration path with evaluation model of node importance degree. These reviews have focused on the skeleton-network reconfiguration of transmission network which aims to establish the main network and restore essential loads. The improvement of the topology structure robustness is usually omitted in the distribution network reconfiguration. Moreover, we would simulate the network reconfiguration in a particular case using these schemes. No reference examines the relationship between node importance degree and system power loss in the process of network reconfiguration.

Network reconfiguration is an importance tool to optimize and control the operation of modern distribution systems. The node importance degree is a significant index to evaluate the practicability of reconfiguration strategy as it reflects the robustness and survivability of network structures after reconstruction. Thus, this paper proposes a novel reconfiguration strategy considering the node importance degree and power loss. To balance the objectives, we formulate a compound objective function which includes two sub-objective functions of minimization of power loss and maximization of node importance degree. And the priority of the indices are determined by the weight coefficients. We employ the quantum particle swarm optimization to address the compound objective reconfiguration problem. Using the proposed strategy, we obtain the reconfiguration configurations which have improved the network economy and robustness. Instead of providing an optimal topology network in detail, this strategy intends to obtain several reconfiguration schemes with better performance as the guidance of dispatching operation. Furthermore, it reveals that the relationship among the evaluating indices. The application examples illustrate that the proposed reconfiguration strategy are more suitable for the practical distribution network compared with other strategies.

The material in this paper is arranged in the following order: section 2 deals with the evaluation method of node importance degree. Section 3 gives the reconfiguration mathematical model and objective function. The procedure of compound objective reconfiguration is presented in section 4. In section 5, we present the simulation results and discussion to assess the effectiveness of the proposed strategy. Conclusion and remarks are highlighted in section 6.

## Node Importance Evaluation Model

Topology structure of a real distribution system can be represented by an abstract graph, whose nodes and branches are corresponding to bus bars and electric elements or switches, respectively. Assuming a system with *n* nodes and *m* switches, the topology graph is described as *G* = (*V*,*E*), where *V* = {*v*_1_,*v*_2_,…*v*_*n*_} represents the set of nodes and *E* = {*e*_1_,*e*_2_,…*e*_*n*_} represents the set of branches. The nodes and branches, as the core elements of the topology graph, are significant to analyze the network connectivity. A branch exists between two nodes if there is a direct link between the associated nodes. Therefore, the network connectivity of the graph *G* = (*V*,*E*) is represented as an adjacency matrix *A* which is expressed as:

$$A={\left(a_{ij}\right)}_{n\times n}=\{\begin{array}{cc} a_{ij}=1 & v_{i}v_{j}\in E\\ a_{ij}=0 & v_{i}v_{j}\notin E \end{array}$$ (1)

Where *a*_*ij*_ is a binary variable, if there is a branch between node *i* and node *j*, then *a*_*ij*_ = 1. Otherwise, *a*_*ij*_ = 0.

The degree of node *i* is defined as the maximum number of branches emanating from the node. It is denoted by *D*_*i*_.

$$D_{i}=\sum_{j=1}^{n}a_{ij}$$ (2)

Generally, the node degree is used to measure the importance of each node in studying complex topology network. It is assumed that the nodes with more branches connected are more important [10]. However, some critical nodes in real network have low node degrees because there are a few branches linked them. The node degree cannot accurately express the importance of these nodes. To address the inconsistency, we introduce the node importance degree to describe the importance of nodes in the network.

The node importance degree reflects the network robustness by assessing the importance of the nodes [25]. In this section, we apply the node contraction to analyze the node importance degree. Let *G* = (*V*,*E*) be an original distribution network. Let *G*′ = (*V*′,*E*′) represent the topological structure after *i*-node contraction. That is, *i*-node and the nodes connected with *i*-node are merging into a new node *i*′. The *i*-node importance degree is formulated as:

$$\delta_{i}=1/\left(n^{'}l_{ave}\right)$$ (3)

Where *δ*_*i*_ represents *i*-node importance degree, *n*′ is the number of nodes in new network *G*′, the average distance among the nodes is *l*_*ave*_. In the new network topology, *l*_*ave*_ is described as:

$$l_{ave}=\sum_{j=1}^{V^{'}}\sum_{k=1}^{V^{'}}\frac{d_{\min,j,k}}{0.5n^{'}(n^{'}-1)}\;(j\neq k)$$ (4)

Where *d*_*min*_ represents the minimum distance between node *j* and node *k*, *V*′ represents the set of nodes in the new network *G*′.

It is observed that *i*-node importance degree *δ*_*i*_ lies on the node degree *D*_*i*_ and its position in network. The original network is changed slightly or remarkably after node contraction. The number of nodes changes from *n* in original network *G* to *n’* in new network *G*′. Similarly, the average shortest distance *l*_*ave*_ between original network and new network is different. Above all, if a node is connected with more branches, namely the larger node degree, its contraction would reduce the number of nodes considerably, the network would contract together much better. For example, only one node remains after the contraction of the center node in the special star network. So the node has large node importance degree. Secondly, if the nodes at pass location are contracted, the average shortest distance would be decreased greatly because these nodes are necessary in the shortest path of many node pairs. We obtain a large node importance degree.

**Fig 1:**
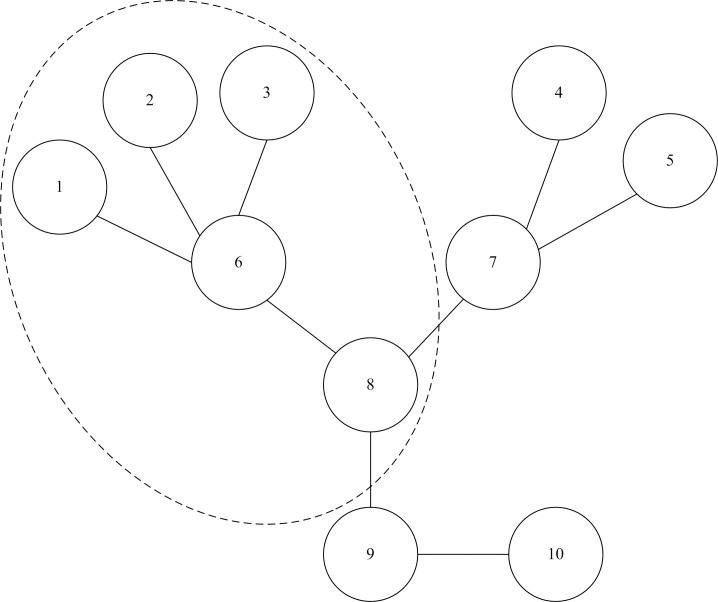
Initial distribution network.

**Fig 2:**
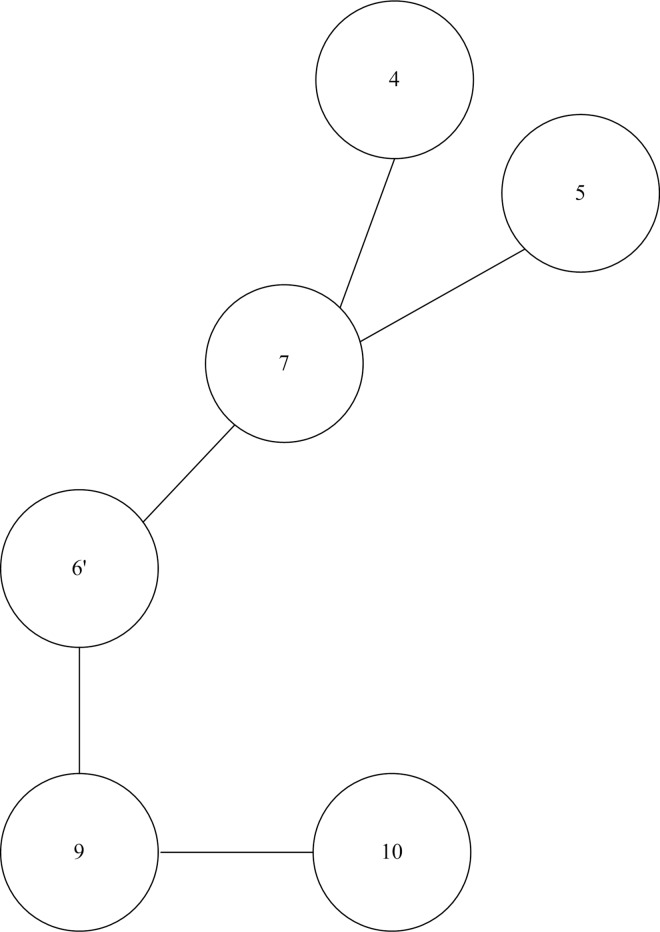
Network after node contraction.

According to the physical definition *δ*_*i*_, the maximum node importance degree obtained is 1 if there is only one node in the network after contraction. In general, the average shortest distance is less than 1 [23]. Therefore, the range of node importance degree is 0 < *δ*_*i*_ ≤ 1. An initial network with 10 nodes and 9 branches is shown in Fig 1. For obtaining the node importance degree *δ*_*i*_ of node 6, we should update the original network after node contraction. It means that the nodes 1, 2, 3, 8 directly connected this node and node 6 would merge into a new node 6’. Fig 2 shows the new network after node contraction. As a result, the calculated node importance degrees of nodes 6, 7, and 8 are given in Table 1.

**Table 1:** Comparison of *δ*_*i*_ and *D*_*i*_ of nodes.

Node	*D*_*i*_	*δ*_*i*_
6	4	0.0781
7	3	0.0652
8	3	0.0833

From the Fig 1, node 8 is the most critical node in the network. But node 8 has lower degree *D*_*i*_ than nodes 6 and 7. It is evident that the node degree cannot accurately identify the importance of some critical nodes. The importance degree of nodes 6, 7 and 8 are 0.0781, 0.0652 and 0.0833, respectively. It is obvious that the node 8 is more important than nodes 7 and 6. The obtained results illustrate that the node with larger node degree may not be more important. Moreover, nodes 7 and 8 have the same node degree (*D*_*i*_ = 3), but node 8 is much more important than node 7 through comparing the *δ*. Thus, the index of node importance degree is more distinct to distinguish network node importance than node degree.

## Compound Objective Function

Distribution network reconfiguration is a nonlinear combinational optimization problem which aims to find the optimal radial configurations of satisfying different optimization objectives and operation constraints. In order to improve network robustness and economy, we select the node importance degree and active power loss as the optimization indices of reconfiguration.

### (1) Node importance degree index

The node importance degree is an evaluation index of network robustness. The importance degree of a node is higher, the node is more important. In the previous section, the maximum node importance degree obtained is 1 and the range is 0 < *δ*_*i*_ ≤ 1. Therefore, the objective function of network robustness is formulated as:

$$f_{\delta }=\max\{1/\delta_{1},1/\delta_{2}\ldots 1/\delta_{n}\}$$ (5)

Where *n* is the number of nodes in the original network, *δ*_*i*_ represents *i-*node importance degree.

### (2) Power loss index

The active power loss of the system is considered as one of the objectives because excessive power loss would increase overheating of the electric components and additional costs. The total power loss of a distribution network is computed by summing the loss of all branches and it should be reduced during reconfiguration. In the modern distribution systems, the switches are divided into sectionalizing switches (normally closed switches) and tie switches (normally open switches). The status of switches determines the corresponding branch connected or not. If the branch is disconnected, the power loss of this branch need not be calculated. A distribution system with *m* nodes, the objective function of the system power loss is expressed as follows.

$$f_{loss}=\sum_{b=1}^{m}k_{b}P_{loss(b,b+1)}$$ (6)

Where *f*_*loss*_ is total active power loss of the system, *m* is the number of nodes, *P*_*loss(b*,*b+1)*_ is the power loss of the branch connecting the node *b* and node *b+1*. *k*_*b*_ is the status of the branch connecting the nodes *b* and *b+1*. *k*_*b*_ = 1 if the branch is closed, and *k*_*b*_ = 0 represents opened the branch.

The system power loss *f*_*loss*_ formula is deduced by the power flow equations [28,29]. We get the power loss of the branch connecting the node *b* and node *b+1*.

$$P_{loss(b,b+1)}=r_{b}\frac{P_{b}^{2}+Q_{b}^{2}}{{\left|V_{b}\right|}^{2}}$$ (7)

Where *P*_*b*_ and *Q*_*b*_ are the active and reactive power flows out of the node *b*. The resistance of the branch between nodes *b* and *b+1* is represented as *r*_*b*_. *V*_*b*_ is voltage magnitude of node *b*. Thus, the system power loss can be represented as:

$$f_{ploss}=\sum_{b=1}^{m}k_{b}P_{loss(b,b+1)}=\sum_{b=1}^{m}k_{b}r_{b}\frac{P_{b}^{2}+Q_{b}^{2}}{V_{b}^{2}}$$ (8)

### (3) Compound objective function

Aiming to reduce power loss and improve the node importance degree, the distribution network is reconfigured. In order to optimize these objectives simultaneously, traditional method is performed by a sum of objective functions [30]. The obtained solution is not suitable for the current situation of the network if the relationship of the objectives is in conflict or competition. In this paper, we assign significance weight coefficients to the objective functions and add them up. They are denoted by *w*_*1*_ and *w*_*2*_. And the relationship between them is *w*_*1*_ + *w*_*2*_ = 1 [31]. Then a compound objective function including double sub-objective functions of node importance degree and system power loss is formulated. The terms of the compound objective are not dimensionally homogenous because they have different statistical pattern and different types of parameters. Also, the variation range of the parameters is much different. We should normalize the sub-objective functions and embed the penalty factors. Since the aim of network reconfiguration is the improvement of the performance of the network, the indices after reconfiguration are better than before reconfiguration. In the abnormal condition, the starting point of distribution network reconfiguration is the initial distribution network structure. Thus, the indices of initial network topology is regarded as the normalized standard value. The two normalized sub objective functions are expressed as:

$$f_{ploss}^{\prime }=\frac{f_{ploss}}{f_{plossin}}$$ (9)

$$f_{\delta }^{\prime }=\frac{f_{\delta }}{f_{\delta in}}$$ (10)

Where $f_{ploss}^{\prime }$, *f*_*ploss*_, and *f*_*plossin*_ are the power loss of normalization, current topology, and initial topology, respectively. $f_{\delta }^{\prime }$, *f*_*δ*_, and *f*_*δin*_ represent the node importance degree of normalization, current topology, and initial topology, respectively.

The network reconfiguration is a mixed nonlinear optimization problem. During the process of optimization, the indices after reconfiguration are better than initial network. To accelerate the speed of optimization, we assign the penalty factors (*x*_*1*_, *x*_*2*_) into the compound objective function. The penalty factors depend on the initial values and current values of the normalized function. For example, if the normalized function $f_{ploss}^{\prime }\geq 1$, it means that the obtained configuration is not suitable because the current power loss is greater than initial network. We should adjust the optimization search direction by setting a large penalty factor. On the other hand, if $f_{ploss}^{\prime }<1$, we reserve the solution and the *x*_*1*_ set 1. So the penalty factors would impact on the speed of the optimization algorithm. The *x*_*1*_ and *x*_*2*_ are described as:

$$x_{1}=\{\begin{array}{cc} N & f_{ploss}^{\prime }\geq 1\\ 1 & other \end{array}$$ (11)

$$x_{2}=\{\begin{array}{cc} N & f_{\delta }^{\prime }\geq 1\\ 1 & other \end{array}$$ (12)

Where *N* is a decimal integer. Therefore, the compound objective function is described as:

$$minf=min(w_{1}x_{1}f_{{}_{ploss}}^{\prime }+w_{2}x_{2}f_{{}_{\delta }}^{\prime })=min(w_{1}x_{1}\frac{f_{ploss}}{f_{plossin}}+w_{2}x_{2}\frac{f_{\delta }}{f_{\delta in}})$$ (13)

Where *w*_1_ and *w*_2_ represent the weight coefficients.

The compound objective function (13) subjects to the network operation constraints, as given by the set of Eqs (14–17).

$$I_{ij}\leq I_{ij.\max}$$ (14)

$$S_{ij}\leq S_{ij.\max}$$ (15)

$$V_{i.\text{min}}\leq V_{i}\leq V_{i.\text{max}}$$ (16)

$$\left|\det(A)\right|=\{\begin{array}{c} 1\\ 0 \end{array}$$ (17)

Where subscripts of *max* and *min* are upper and lower bounds, *A* is bus incidence matrix and is calculated by Eq (1). If *det*(*A*) = 1 means that the identified topology structure is a radial configuration. The Eqs (14) and (15) represent current thermal constraints and transmission power capacity constraints at each branch. The Eq (16) represents the nodal voltage constraints.

## Methodology

**Fig 3:**
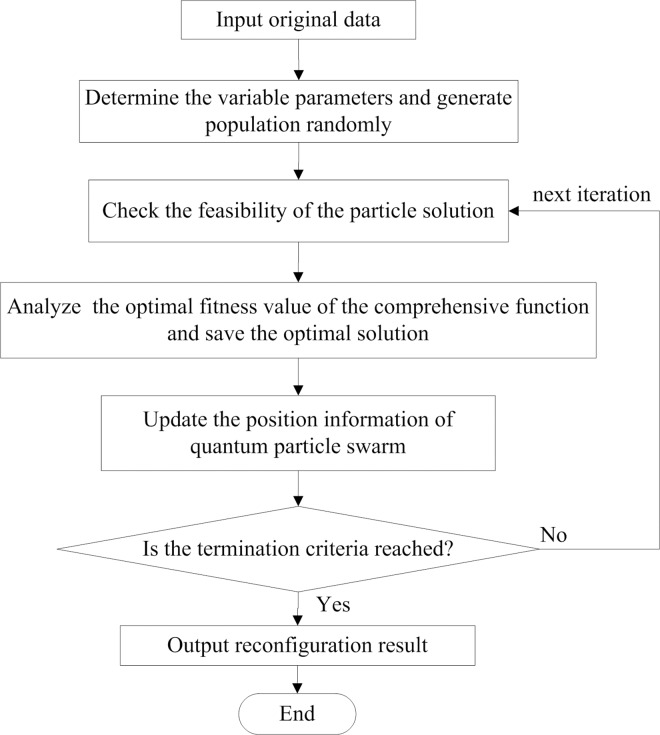
Flow of the network reconfiguration.

In this paper, we propose a compound objective reconfiguration strategy for distribution networks. Under the operation constraint, the network reconfiguration aims to find the topologies which satisfies the compound objective. It is a complicated combinatorial optimization problem, making the use of classical methods to solve it optimally prohibitive. This study employs the quantum particle swarm optimization (QPSO) to address the reconfiguration problem as it has the capacity to parallel optimize combinational functions. And the QPSO has been successfully to solve the optimization problems in electrical engineering [32–34]. The QPSO is a population-based evolutionary technique that has many key advantages over other optimization algorithms [35–36]. It can hold the best solution of each particle and avoid premature convergence. Furthermore, it is less sensitive to the nature of the objective function and has ability to escape the local minima. A general structure of the QPSO is described as Fig 3.

(1) Input initial data. The inputs of the procedure are initial topology structure, population size, compound objective function, and maximum iterations. Then we calculate the initial values of node importance degree and power loss.

(2) Design variable expression and code scheme. A good variable expression is critical to improve the efficiency of optimization algorithm. A solution is considered as a particle. Assuming that a solution vector is *x* = (*x*_1_,*x*_2_…*x*_*n*_), where *n* is the number of bits of a particle or the number of the tie switches, *x*_*n*_ represents the label of opened switch. For a distribution network with *n* tie switches, the number of fundamental loops is *n*. The number of bits of each particle is equal to the number of loops. Each bit indicates the branch number, located in the corresponding loop, that should be open. We obtain the each variable range by analyzing the set of each fundamental loop vector. In each loop, we randomly select a switch to open. Thus, a set of random candidate solutions is established.

(3) Check the validity of the solutions. A solution is corresponding to a possible topology. Since the distribution system operates with a radial topology, we have to check the feasible of the candidate solutions. The bus incidence matrix *A* of a candidate topology is generated according to Eq (1). The determinant absolute value of matrix *A* is represented as |*det*(*A*)|. The current topology is radial if |*det*(*A*)| = 1, then continue the next step. Else if the determinant of matrix *A* is equal to zero, this means that either the current topology is not radial or group of power loads are disconnected from the service. We reexamine the feasibility of next group of particles in the population.

(4) Set the weight coefficients (*w*_*1*,_ *w*_*2*_). And the fitness value *f* of an effective particle is calculated by $f=w_{1}x_{1}f_{{}_{ploss}}^{\prime }+w_{2}x_{2}f_{{}_{\delta }}^{\prime }$.

(5) Calculate the fitness value *f* of each particle. In *k* iteration generation, $P_{i}^{k}$ and $P_{g}^{k}$ are the current position and global best position of particle *i*. The optimal position of *D*-dimensional particle *i* is $P_{ido}^{k}=\varphi P_{id}^{k}+(1-\varphi )P_{gd}^{k}$. And the global average position $P_{av}^{k}$ of each particle is obtained by the equation of $P_{av}^{k}=\frac{1}{N}\sum_{i=1}^{N}P_{i}^{k}$.

(6) Update the position vectors of each particle in the population using the equation of $X_{id}^{k+1}=round(P_{ido}^{k}\pm \beta *|P_{av}^{k}-X_{id}^{k}|*ln(1/u))$, where *u* and *φ* are the uniformly distributed random number in [0,1], *round*() is the integral function, and *β* is used to control the convergence speed of the algorithm.

(7) Repeat steps (4)–(6) until a termination criterion is satisfied. The obtained *X*_*id*_ is the best solution of the network. Then output the optimal position.

## Results and Discussion

### Case I: 33-bus system

IEEE33-bus system broadly published in literatures is a standard 12.66kV network, having 33 nodes, 32 sectionalizing switches and 5 tie-switches. It is employed to demonstrate the effectiveness of the proposed strategy. The system details are given in [34]. The normally closed switches are 1 to 32, and normally open switches are 33 to 37. The active and reactive power loads of the system are 3715kW and 2300kVar, respectively. This paper formulates the distribution network reconfiguration as a compound objective optimization problem. Quantum particle swarm optimization is used to solve this problem. The population size and the maximum number of iterations are 50 and 200, respectively.

The original importance degree of each node is computed by using node contraction method. In order to describe and evaluate the relative role of each node, the original importance degrees should be normalized further into node relative importance degree. The normalized standard value is ideal maximum *δ*_*i*_ if the index of node importance degree is considered as the only reconfiguration objective. Under this circumstance, the each node importance degree obtained after reconfiguration is shown in Table 2.

**Table 2:** Ideal node importance degree (after reconfiguration).

Node	*δ*_*i*_	Node	*δ*_*i*_	Node	*δ*_*i*_
1	0.0053	12	0.0050	23	0.0053
2	0.0053	13	0.0050	24	0.0050
3	0.0056	14	0.0051	25	0.0050
4	0.0055	15	0.0057	26	0.0055
5	0.0055	16	0.0054	27	0.0055
6	0.0061	17	0.0054	28	0.0055
7	0.0058	18	0.0054	29	0.0057
8	0.0061	19	0.0050	30	0.0054
9	0.0058	20	0.0051	31	0.0051
10	0.0051	21	0.0054	32	0.0051
11	0.0049	22	0.0051	33	0.0054

Table 2 presents the obtained *δ*_*i*_ of optimal configuration by optimizing the single objective of node importance degree. It can be seen that the maximum node importance after reconfiguration is nodes 6 and 8 with 0.0061. Note that this constant value is supposed as the standard value, the node importance degree rank is regulated to 1. Therefore, the relative node importance degrees before and after reconfiguration are listed in Table 3.

**Table 3:** Relative node importance degree.

	Node importance degree				Node importance degree		
Node	Initial	Reconfiguration	Improving	Node	Initial	Reconfiguration	Improving
1	0.6393	0.8689	35.91%	18	0.6557	0.8852	35.00%
2	0.6885	0.8689	26.20%	19	0.6712	0.8197	21.96%
3	0.7213	0.9180	27.27%	20	0.6712	0.8361	24.40%
4	0.7049	0.9016	27.90%	21	0.6721	0.8852	31.71%
5	0.7049	0.9016	27.90%	22	0.6393	0.8361	30.78%
6	0.7377	1.0000	35.56%	23	0.6557	0.8689	32.51%
7	0.7049	0.9508	34.88%	24	0.6557	0.8197	25.01%
8	0.7049	1.0000	41.86%	25	0.6393	0.8197	28.22%
9	0.7049	0.9508	34.88%	26	0.6721	0.9016	34.15%
10	0.7049	0.8361	18.61%	27	0.6721	0.9016	34.15%
11	0.7049	0.8033	13.96%	28	0.6721	0.9016	34.15%
12	0.7049	0.8197	16.29%	29	0.6721	0.9344	39.03%
13	0.7049	0.8197	16.29%	30	0.6721	0.8852	31.71%
14	0.7049	0.8361	18.61%	31	0.6721	0.8361	24.40%
15	0.7049	0.9344	32.56%	32	0.6721	0.8361	24.40%
16	0.7049	0.8852	25.58%	33	0.6393	0.8852	38.46%
17	0.7049	0.8852	25.58%				

Following the steps presented of the reconfiguration strategy, we obtain a set of best solutions by adjusting weight coefficients. The simulation results with the values of the objective functions are shown in Table 4.

**Table 4:** Reconfiguration results of different weight coefficients (33-bus system).

Weight coefficients	Open switches	Power loss(kW)	$\sum_{i}^{n}\delta_{i}$
Initial network	33 34 35 36 37	202.6795	22.5082
*w*_1_ = 1,*w*_2_ = 0	7 14 9 32 37	139.4410	23.8197
*w*_1_ = 0.9,*w*_2_ = 0.1	7 14 9 32 28	139.8705	25.0328
*w*_1_ = 0.8,*w*_2_ = 0.2	7 14 9 32 28	139.8705	25.0328
*w*_1_ = 0.7,*w*_2_ = 0.3	7 14 9 32 28	139.8705	25.0328
*w*_1_ = 0.6,*w*_2_ = 0.4	7 14 10 32 28	140.5971	25.2787
*w*_1_ = 0.5,*w*_2_ = 0.5	7 14 10 32 28	140.5971	25.2787
*w*_1_ = 0.4,*w*_2_ = 0.6	7 14 10 32 28	140.5971	25.2787
*w*_1_ = 0.3,*w*_2_ = 0.7	7 14 10 32 27	143.9131	25.5902
*w*_1_ = 0.2,*w*_2_ = 0.8	5 14 11 32 27	167.1668	26.4918
*w*_1_ = 0.1,*w*_2_ = 0.9	5 13 11 32 27	174.3956	26.7541
*w*_1_ = 0,*w*_2_ = 1	18 12 11 31 24	304.2476	28.9344

To validate the effectiveness of the proposed reconfiguration model, the obtained extreme fitness values are compared with the results of other methods by optimizing each objective function separately that are shown in Tables 5 and 6, respectively. In the compound function model, the weight vector is (*w*_1_ = 0, *w*_2_ = 1) when only the maximization of node importance degree is considered as the reconfiguration objective. And the weight vector is equal to (*w*_1_ = 1, *w*_2_ = 0) if the reconfiguration objective is the minimization of system power loss.

**Table 5:** Obtained results by optimizing power loss as the only objective.

Methods	Open switches	Power losses(kW)	Saving
Proposed method	7 14 9 32 37	139.4410	31.22%
Genetic algorithm[17]	7 14 9 32 37	139.5330	31.08%
DPSO[34]	7 14 9 32 37	139.4410	31.22%
Heuristic approach[36]	7 14 9 32 37	140.2634	30.73%
Minimum-Current[37]	7 14 9 32 37	139.5510	31.15%

**Table 6:** Obtained results by optimizing node importance degree as the only objective.

Methods	Open switches	$\sum_{i}^{n}\delta_{i}$	Improving
Proposed method	18 12 11 31 24	28.9344	30.92%
Genetic algorithm[17]	7 14 9 32 37	—	—
DPSO[34]	7 14 9 32 37	—	—
Heuristic approach[36]	7 14 9 32 37	—	—
Minimum-Current[37]	7 14 9 32 37	—	—

Table 5 shows the results when the minimization of power loss is considered as the only objective function. It is obvious that the compound objective reconfiguration strategy is capable of finding the best configuration which is comparable to other methods. The generated best switches combination [7 14 9 32 37] represents a topology network in which all the switches are closed except the sectionalizing switches 7, 14, 9, 32, and 37. By comparing to the Tables 4 and 5, the power loss of final configuration obtained is 139.4410kW which is less than other approaches in [17, 36, 37]. Table 6 indicates that only our strategy can reach the optimal solution in optimizing the objective of the maximization of node importance degree. It can be seen that the opened switches combination is [18 12 11 31 24] which is different in references [17, 34, 36, 37]. Having found this final solution, the relative importance degree of each node is shown in Table 2. As shown, the importance degrees of all nodes have been improved after reconfiguration. And the sum of relative node importance degree has reached the maximum value 28.9344 which is increased by 30.92% compared to the initial network. Specially, The relative importance degree of 29-node after reconfiguration is increased by 39.03% of its initial value. Thus, the network robustness has been improved significantly after reconfiguration. However, it is not favorable for economic running because the power loss is higher 50.37% than initial network.

**Fig 4:**
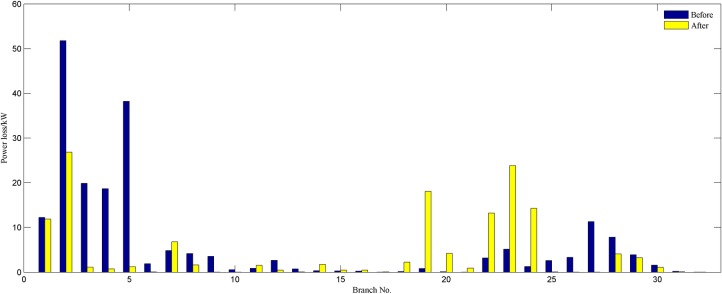
Each branch power loss of 33-bus system.

**Fig 5:**
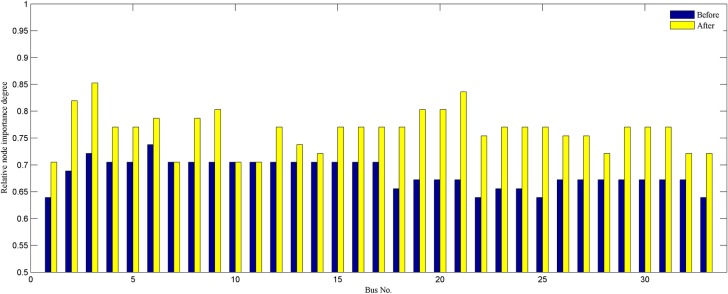
Each node importance degree of 33-bus system.

In order to verify the performance of the proposed strategy, we analyze statistically the reconfiguration results of different weight coefficients. By comparing the results obtained in Tables 3–6, it is noticeable that the proposed model is more efficient and flexible in terms of candidate reconfiguration schemes. We get a set of radial configurations of improving the network economy and robustness by adjusting weight coefficients. The decision maker can change flexibly the operation configuration according to the actual operation requirements and his experience. Hence, the proposed strategy is more suitable for the actual distribution system reconfiguration than conventional methods. To compare the change of the reconfiguration objective before and after network reconfiguration, we provide the obtained reconfiguration results when *w*_1_ = *w*_2_ = 0.5. The best configuration identified is the following: 33–7, 34–14, 35–10, 36–32, and 37–28. Fig 4 clearly shows each branch’s active power loss of the best configuration and the initial network. This amount is 140.5971kW which is approximately 30.4% reduction of initial power loss. A comparison of each node’s importance degree for both networks is presented in Fig 5. It is observed that each node importance degree of the best configuration is improved. As it can be gathered from the figure, the sum of nodes importance degree is 25.2787, which is higher than the initial network.

The weight coefficients represent the weight of each sub-objective index system. If *w*_1_ is greater than *w*_2_, the sub-objective of power loss takes priority over the node importance degree. On the contrary, when *w*_1_ < *w*_2_, the node importance degree is considered as a dominated objective. In order to investigate the relation of the objectives, weight coefficients for maximum tardiness is decreased by 0.1, and weight coefficients for maximum completion time is added by 0.1 in ascending. Table 4 gives the obtained results of different scenarios network reconfiguration. We can derive that the weight coefficients would influence the changes of the network economy and robustness. For example, in situation *A* (*w*_*1*_ *=* 0.3, *w*_*2*_ *=* 0.7), the best configuration obtained is 7, 14, 10, 32, and 27, where the power loss and the sum of node importance degree are 143.9131kW and 25.5902, respectively. The optimal power loss of situation *B* (*w*_*1*_ *=* 0.2, *w*_*2*_ *=* 0.8) is 167.1668kW which is greater than 143.9131kW. The sum of node importance degree increases to 26.4918. Compared the two solutions, an investment cost of the situation *A* is higher than situation *B*. But the network robustness of situation *B* are more attractive than situation *A*. Based on these results, it is possible to say that increment of node’s importance degree could increase the power loss (solutions listed in the third and fourth rows of Table 4). It means that the improvement of network robustness may reduce the network economy. The two sub-objectives are in conflict but not in simple inverse linear relation.

We can observe that the different weight coefficients would lead to the same reconfiguration results from Table 4. Under the circumstances, it is illustrated that the reconfiguration results are less sensitive to the weight coefficients. We can select the final topology by fixing randomly a set of weight vector. In Table 4, the scenarios of weight vectors adopted *w =* (0.9,0.1), (0.8,0.2), and (0.7,0.3) lead to the same experimental results. A group of *w* = (0.9,0.1) is selected at random, the final reconfiguration results are described as Table 7.

**Table 7:** Final Reconfiguration results (33-bus system).

Weight coefficients	Open switches	Power loss(kW)	$\sum_{i}^{n}\delta_{i}$
*w*_1_ = 1,*w*_2_ = 0	7 14 9 32 37	139.4410	23.8197
*w*_1_ = 0.9,*w*_2_ = 0.1	7 14 9 32 28	139.8705	25.0328
*w*_1_ = 0.5,*w*_2_ = 0.5	7 14 10 32 28	140.5971	25.2787
*w*_1_ = 0.3,*w*_2_ = 0.7	7 14 10 32 27	143.9131	25.5902
*w*_1_ = 0.2,*w*_2_ = 0.8	5 14 11 32 27	167.1668	26.4918
*w*_1_ = 0.1,*w*_2_ = 0.9	5 13 11 32 27	174.3956	26.7541
*w*_1_ = 0,*w*_2_ = 1	18 12 11 31 24	304.2476	28.9344

### Case II: 69-bus system

**Fig 6:**
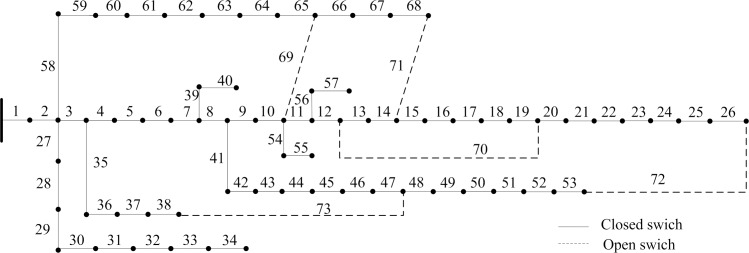
Initial topology of the 69-bus system.

The next case is PG&E 69-bus system which is also employed to demonstrate the feasibility and effectiveness of the proposed strategy. It is comprised of 68 sectionalizing switches and 5 tie switches. The initial topology is shown in Fig 6 and the system data is given in [36]. We establish a compound objective function as the reconfiguration objective. The initial parameters of the proposed strategy are the same of the case I. The experimental results obtained in different weight vectors are shown in Table 8.

**Table 8:** Comparison of reconfiguration results before and after reconstruction (69-bus system).

*w*_*1*_,*w*_*2*_	Open switches	Power loss (kW)	Saving	$\sum_{i}^{n}\delta_{i}$	Minimum importance degree	Improving
Initial	69 70 71 72 73	224.9654	—	51.5625	0.6875	—
(1,0)	69 70 14 50 44	99.6205	55.96%	45.5000	0.6250	-9.09%
(0.9,0.1)	69 18 13 52 46	108.8558	51.87%	53.9698	0.7480	4.67%
(0.8,0.2)	69 18 14 52 46	109.2416	51.70%	54.7394	0.7500	6.16%
(0.7,0.3)	63 18 14 52 47	119.3112	47.25%	62.0985	0.8750	20.43%
(0.6,0.4)	63 18 14 52 47	119.3112	47.25%	62.0985	0.8750	20.43%
(0.5,0.5)	63 17 14 52 47	120.4869	46.73%	62.6643	0.8750	21.53%
(0.4,0.6)	62 16 71 52 47	124.3264	45.03%	63.9375	0.8750	23.97%
(0.3,0.7)	62 16 71 52 47	124.3264	45.03%	63.9375	0.8750	23.97%
(0.2,0.8)	62 16 71 52 47	124.3264	45.03%	63.9375	0.8750	23.97%
(0.1,0.9)	62 16 71 52 47	124.3264	45.03%	63.9375	0.8750	23.97%
(0,1)	61 16 71 52 47	125.5759	44.48%	64.2500	0.8750	24.61%

Similarly to verify the results presented in Table 8, the extreme points of the obtained best configuration are compared with the results of other methods by optimizing only the objective of minimum power loss that are shown in Table 9. The results presented demonstrate that the proposed model is capable of finding best solution. The power loss after reconfiguration is amount 55.72% of initial real power loss, i. e. 99.6205kW, which is comparable to [36] and [37] but less than other methods. It is again implied the effectiveness of the proposed method in terms of searching optimal reconfiguration network. Moreover, the network robustness would be reduced because the minimum relative node importance degree is reduced by 9.09% compared to the original network. Since there are no references analyzing node importance degree, the proposed model has not been compared with other schemes in view of the node importance degree index.

**Table 9:** Obtained results by optimizing power losses as the only objective (69-bus system).

Methods	Open switches	Power loss (kW)	Saving
Proposed method	69 70 14 50 44	99.6205	55.72%
Genetic algorithm[17]	69 70 14 50 44	100.9395	55.12%
DPSO[34]	69 70 14 50 44	99.6205	55.72%
Heuristic approach[36]	69 70 14 50 44	99.8306	55.62%
Minimum-Current [37]	69 70 14 50 44	99.6205	55.72%

**Fig 7:**
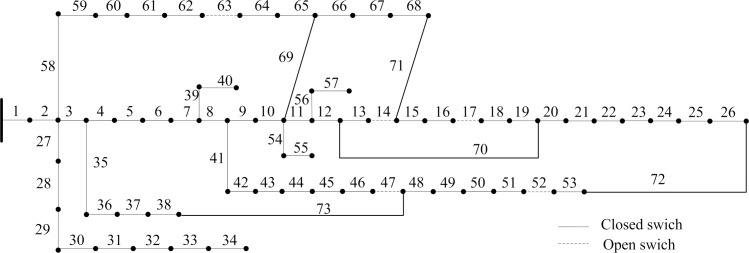
Topology network of 69-bus system after reconfiguration.

**Fig 8:**
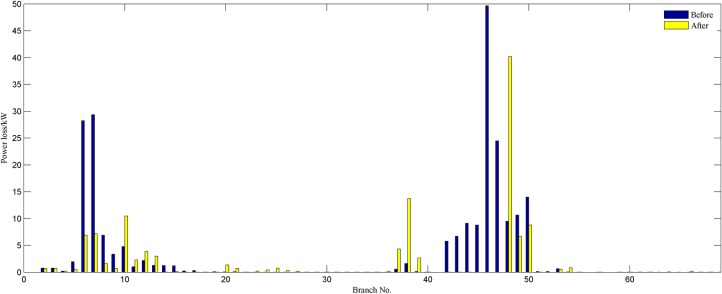
Each branch power loss of 69-bus system.

**Fig 9:**
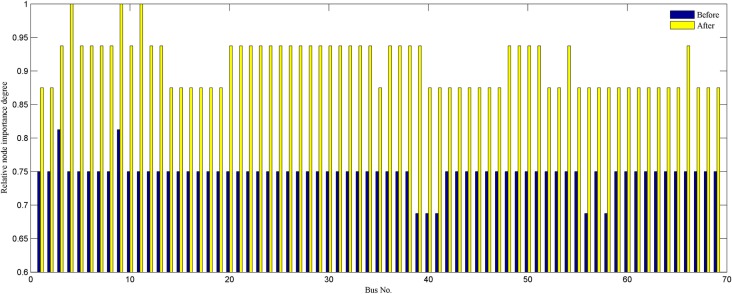
Each node importance degree of 69-bus system.

The solution set, that is, the results obtained by applying compound reconfiguration objective function to 69-bus system reconfiguration are presented in Table 8. Once the weight vector is determined, the corresponding optimal solution would be generated. The obtained reconfigurations by modelling different scenarios network reconfiguration have improved the network robustness and economy. Thus, the proposed strategy is able to present more benefit to utility operator because of providing several high-quality schemes. Assuming that the minimum power loss is just as important as the improvement node importance, the weight vector is *w =* (0.5,0.5). The initial network is modified by closing the opened switches combination [69 70 71 72 73] and opening closed switches combination [63 17 14 52 47] to represent best topology which is shown in Fig 7. Fig 8 compares each branch’s power loss profiles both initial and optimal topologies. This amounts to a reduction of 46.73% in total power loss. And each node’s relative node importance degree is depicted in Fig 9. It is observed that minimum node importance degree for this modified configuration is increased to 0.8750. This shows the improvement of network robustness using the proposed reconfiguration model. Moreover, the enhancement of the node importance degree could harm the index of power loss minimization by analyzing the results of the third and fifth columns.

By observing the results represented in Table 8, the proposed strategy is more efficient and flexible because a set of candidate optimal configurations are appeared. Also it is observed that different weight vectors lead to same configuration. In this circumstance, we select randomly a set of weight vector. Table 10 summarizes the final reconfiguration results.

**Table 10:** Final reconfiguration results (69-bus system).

*w*_*1*_,*w*_*2*_	Open switches	Power loss (kW)	Saving	$\sum_{i}^{n}\delta_{i}$	Minimum importance degree	Improving
Initial	69 70 71 72 73	224.9654	—	51.5625	0.6875	—
(1,0)	69 70 14 50 44	99.6205	55.96%	45.5000	0.6250	-9.09%
(0.9,0.1)	69 18 13 52 46	108.8558	51.87%	53.9698	0.7480	4.67%
(0.8,0.2)	69 18 14 52 46	109.2416	51.70%	54.7394	0.7500	6.16%
(0.7,0.3)	63 18 14 52 47	119.3112	47.25%	62.0985	0.8750	20.43%
(0.5,0.5)	63 17 14 52 47	120.4869	46.73%	62.6643	0.8750	21.53%
(0.4,0.6)	62 16 71 52 47	124.3264	45.03%	63.9375	0.8750	23.97%
(0,1)	61 16 71 52 47	125.5759	44.48%	64.2500	0.8750	24.61%

## Conclusions

This paper presents a new reconfiguration strategy with compound objective function. Based on the complex theory, the quantitative node importance degree is an effective index of distribution network reconfiguration because it reflects the network robustness by evaluating the node importance. To evaluate the reconstructed network’s economy and robustness, we build a compound objective function which incorporates with minimization of power loss and maximization of node importance degree. Then the quantum particle swarm algorithm is used to solve this compound objective optimization problem. Simulation results and the performance assessment analysis illustrate that the effectiveness of the proposed reconfiguration strategy. By adjusting weight coefficients, the proposed strategy intends to obtain a set of best configurations of improving the network economy and robustness. Thus, the obtained solutions bring about an extra flexibility for decision maker. They can choose flexibility the appropriate plans in accordance with the operation condition and different requirements of the system. It will benefit the guidance of dispatching operation.
